# Correction to ‘Seroprevalence Screening of Chronic Aspergillus Infection in a Post‐Tuberculosis Cohort in Senegal: A Cross‐Sectional Study Comparing ELISA and Rapid Diagnostic Tests’

**DOI:** 10.1111/myc.70202

**Published:** 2026-06-23

Mariama T. , N. I. Mbaye , S. Djiby , et al., “Seroprevalence Screening of Chronic Aspergillus Infection in a Post‐Tuberculosis Cohort in Senegal: A Cross‐Sectional Study Comparing ELISA and Rapid Diagnostic Tests,” Mycoses 69, no. 4 (2026): e70174, 10.1111/myc.70174.41964461 PMC13069597

The first and last names of the authors were inadvertently swapped and have been corrected.

The correct author names are: Touré M, Ndiaye IM, Sow D, Diallo MA, Diongue K, Seck MC, Ndiaye M, Ndiaye D, Diop NF, Fortes L, Denning DW, Badiane AS.

The online version of this article has been corrected accordingly.

We apologize for this error.

